# Plasticity and Formability of Annealed, Commercially-Pure Aluminum: Experiments and Modeling

**DOI:** 10.3390/ma13194285

**Published:** 2020-09-25

**Authors:** Jinjin Ha, Johnathon Fones, Brad L. Kinsey, Yannis P. Korkolis

**Affiliations:** 1 Department of Mechanical Engineering, University of New Hampshire, 33 Academic Way, Durham, NH 03824, USA; jinjin.ha@unh.edu (J.H.); johnathon.fones@unh.edu (J.F.); brad.kinsey@unh.edu (B.L.K.); 2 Department of Integrated Systems Engineering, The Ohio State University 1971 Neil Avenue, Columbus, OH 43210, USA

**Keywords:** aluminum, plastic anisotropy, hardening curve, formability, forming limit diagram, digital image correlation, finite element analysis

## Abstract

The plasticity and formability of a commercially-pure aluminum sheet (AA1100-O) is assessed by experiments and analyses. Plastic anisotropy of this material is characterized by uniaxial and plane-strain tension along with disk compression experiments, and is found to be non-negligible (e.g., the r-values vary between 0.445 and 1.18). On the other hand, the strain-rate sensitivity of the material is negligible at quasistatic rates. These results are used to calibrate constitutive models, i.e., the Yld2000-2d anisotropic yield criterion as the plastic potential and the Voce isotropic hardening law. Marciniak-type experiments on a fully-instrumented hydraulic press are performed to determine the Forming Limit Curve of this material. Stereo-type Digital Image Correlation is used, which confirms the proportional strain paths induced during stretching. From these experiments, limit strains, i.e., the onset of necking, are determined by the method proposed by ISO, as well as two methods based on the second derivative. To identify the exact instant of necking, a criterion based on a statistical analysis of the noise that the strain signals have during uniform deformation versus the systematic deviations that necking induces is proposed. Finite element simulation for the Marciniak-type experiment is conducted and the results show good agreement with the experiment.

## 1. Introduction

Realizing the Industrial Internet of Things (IIoT) in stamping has to revolve around three thrusts: sensing, decision-making, and actuating. In comparison to conventional stamping, the material that is processed by such a flexible system may undergo more complex strain-path changes. Hence, predicting its behavior during IIoT-enabled stamping, e.g., its forming limits, and as rapidly as possible, becomes vital. Several assessment methods to obtain the forming limit curve (FLC) of the material, i.e., to identify the strain combinations for safe plastic deformation, have been developed. The concept of FLC was first introduced by Keeler and Backofen [1] and Keeler [2,3] to represent such critical major and minor principal strains, and it was extended into a standard form by Goodwin [4], including the negative minor strain range.

The earliest approach for theoretical calculation of the FLC was based on the tensile instability using bifurcation analysis by Hill [5]. This criterion determines localized necking as an instability under proportional loading, and it only appears in the left hand quadrant of the forming limit diagram (FLD). Swift [6] associated the occurrence of diffuse necking with the maximum load so that it could be applicable for the right hand quadrant of the FLD. Hora et al. [7,8] later developed the Modified Maximum Force Criterion (MMFC) on the basis of a zero extension [9,10].

Marciniak and Kuczyński [11,12] developed a model (widely known as the M-K model) by introducing an imperfection, characterized as a region thinner than the rest of the sheet by a ratio, $f={\mathrm{thick}}_{\mathrm{imperfection}}/{\mathrm{thick}}_{\mathrm{sheet}}$, oriented perpendicular to the major principal stress direction. Force equilibrium and compatibility conditions at the imperfection boundary with the sheet are imposed to calculate the strain ratio increments inside the imperfection and the rest of the sheet. Once this incremental ratio exceeds a critical value, typically 10, necking is assumed to have occurred and the corresponding major and minor principal strains are taken as a point on the FLC. Later, more intensive studies on the imperfection demonstrated that the zero extension direction inside the imperfection would not be consistently perpendicular to the major principal direction and rather rotate during deformation [13,14,15,16]. This allowed the prediction of the FLC for right minor strain values.

Although the original M-K model was based on normal anisotropy, Barlat [17] first showed the influence of orthotropic anisotropy on the FLC using different yield criteria incorporated in the M-K analysis and concluded that the rotation of the principal stress direction with respect to the material orthotropy should be considered in the FLC. In this regard, many of yield criteria developed in the last decade have been widely used to predict the limit strains in the M-K model, such as Hill’93 [18,19], BBC [20,21], and Cazacu-Barlat [22,23,24]. Cao [13,25] investigated the effect of strain path changes on the FLC using the Karafillis and Boyce yield criterion [26]. In addition, the Yld2000-2d yield criterion [27] has been widely used: Aretz [28] adopted it into the M-K model to examine the influence of the plastic anisotropy at a biaxial state on the FLC; FLD for a multi-layered sheet [29]; for high strength steels [30,31,32,33]; and for aluminum sheets [33,34,35]. Path dependence was also investigated by combining anisotropic strain-hardening models [35,36,37,38,39].

In addition to the theoretical prediction of the FLC, accurate experimental characterization of forming limit strains has been of great interest. Keeler [40] measured the principal strains before and after deformation based on a small circle grid pattern etched on the surface of the deforming sheet. Since then, many efforts have been paid to systemize a detection method for limit strains to improve the consistency of the experimental data and exclude the subjective assessment of a human observer. Takashina et al. [41] first proposed the three-circle method in which the limit strains were calculated by the elongations of three overlapped circles near the fracture. In 1972, Hecker [42] developed a method to determine the major and minor strains from three types of ellipses near the fracture and interpreting them into a FLC, which has seen extensive use due to its simplicity. Bragard et al. [43] presented an interpolation method approximating the limit strains at the fracture from a parabolic fitted curve. Later, it was further improved, and the ISO 12004 standard “Metallic materials – sheet and strip—Determination of the forming limit curves” [44] was established based on that methodology.

As a full-field optical method which allows *in-situ* strain measurement based on digital processing of images, i.e., Digital Image Correlation (DIC), has been developed, new methods to determine limit strains have been proposed. Marron et al. [45] suggested a new necking criterion based on the strain rate change with respect to time. The beginning of necking can be determined by the intersection of two linear fitted lines to the strain rate before and after the drastic change. This method proved its robustness and reliability by several researches [46,47], but there is still a concern on the linear fitting especially in the range after the drastic increase of the strain rate. Similar approach was considered by Volk and Hora [48] and Merklein [49,50], in which the FLC was determined by considering the strain-rate. An algorithm was proposed for detecting the critical deformation state based on the time-derivative of the thinning strain-rate. Huang [51] proposed a criterion of the second time-derivative of strain and the identified FLC for AHSSs, which showed good agreement with ISO method. The maximum in the second derivative has also been used by Banabic and co-workers [52], Jain and co-workers [53,54], Safdarian et al. [55], and Ju et al. [56]. Bagheriasl and Worswick [57] used the images obtained at the end of the FLC experiment and two criteria (sharp increase of major strain between images, or diminishing minor strain-rate) to assess the formability of AA3003 brazing sheet at elevated temperatures. They compared these results to circle grid analysis. Magrinho et al. [58] determined the FLC by strain-rate and strain-force evolution of DIC measurement for thin-walled tubes. Finally, Iadicola [59] presented a detailed analysis of the effect of DIC parameters on the accuracy of the displacement and strain fields acquired.

In this study, the plasticity and formability of commercially-pure aluminum is presented. The first part of the study describes experiments on the plastic anisotropy of this material. These experiments inform a constitutive modeling framework that involves a non-quadratic yield function (Yld2000-2d) and a saturation-type hardening curve (Voce). In the second part, FLC experiments using the Marciniak technique are described. Three different methods of experimentally assessing the FLC using DIC are discussed. As a verification of the constitutive modeling performed, finite element (FE) models of the FLC experiments are developed, and their results are compared to the experiments.

## 2. Plastic Anisotropy

### 2.1. Material

**Figure 1:**
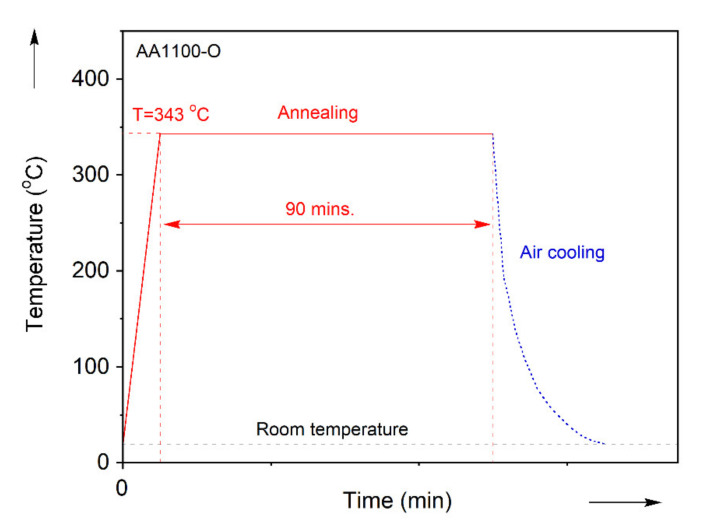
Heat-treatment profile of commercially-pure aluminum.

The material of this study is cold-rolled, commercially-pure aluminum AA1100, received in the -H24 temper as a sheet of 0.5 mm thickness. To restore the formability, the sheets are heat-treated at 343 °C for 90 min, and let to air-cool, see Figure 1. This produced the fully-annealed, -O temper [60,61,62].

### 2.2. Experiments

**Figure 2:**
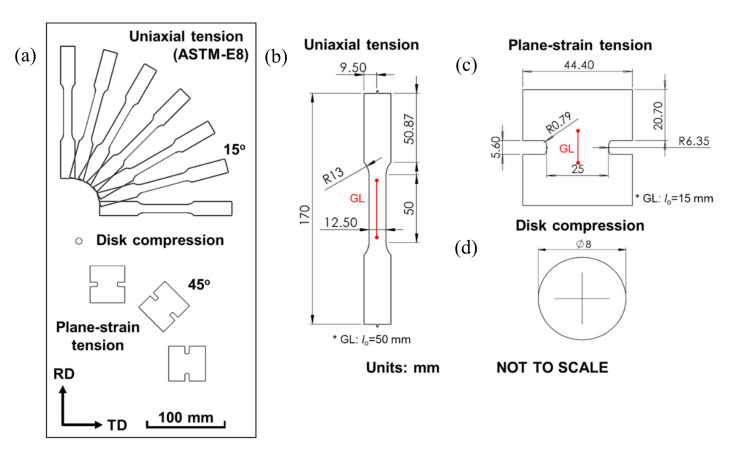
(**a**) Schematic layout of specimens used in this work, on a rolled sheet. Engineering drawings for (**b**) uniaxial tension, (**c**) plane-strain tension and (**d**) disk compression. Initial gauge length (GL) for extensometer is presented in red.

The plastic anisotropy of the AA1100-O sheet sample is measured using three different types of experiments: uniaxial tension in every 15° from the rolling direction (RD), plane-strain tension in every 45° from the RD, and disk compression in the normal direction as seen schematically in Figure 2a. The uniaxial tension experiment is performed using ASTM-E8 specimen, while the plane-strain tension and disk compression experiments use custom designed specimens, with the face in the RD-TD plane and the height along the sheet thickness, from former studies [63,64]. The exact specimen geometries are shown in Figure 2b. The specimens are produced by waterjet machining, and then the edges are lightly polished by hand using sand paper.

For the uniaxial tension and plane-strain tension experiments, a ZwickiLine Universal testing machine (Ulm, Germany) with 5 kN load capacity is used. The crosshead is controlled with a constant displacement rate to impose quasistatic plastic deformation in the specimen: The rates of 0.1 mm/s for the uniaxial tension and 0.025 mm/s for the plane-strain tension are applied to induce a strain rate of 10^−3^ /s in the test-section. During the experiments, 2D Digital Image Correlation (2D-DIC) is used, with images acquired by a Blackfly USB3 5.0 MP (BFLY-U3-50H5C-C, FLIR, Wilsonville, OR, USA) digital camera with 2448 × 2048 pixel resolution using the Point Grey FlyCapture software. The VIC-2D DIC image post-processing software (Correlated Solutions, S.C., USA) is then used to calculate the surface strain field, using a subset of 29, step-size of 7, and filter of 15 pixels for the uniaxial tension and 9, 1 and 15, respectively, for the plane-strain tension. Each experiment is conducted thrice to confirm the test repeatability. Since these repetitions are found to be very consistent, only one result from each case is shown in the paper.

#### 2.2.1. Uniaxial Tension

**Figure 3:**
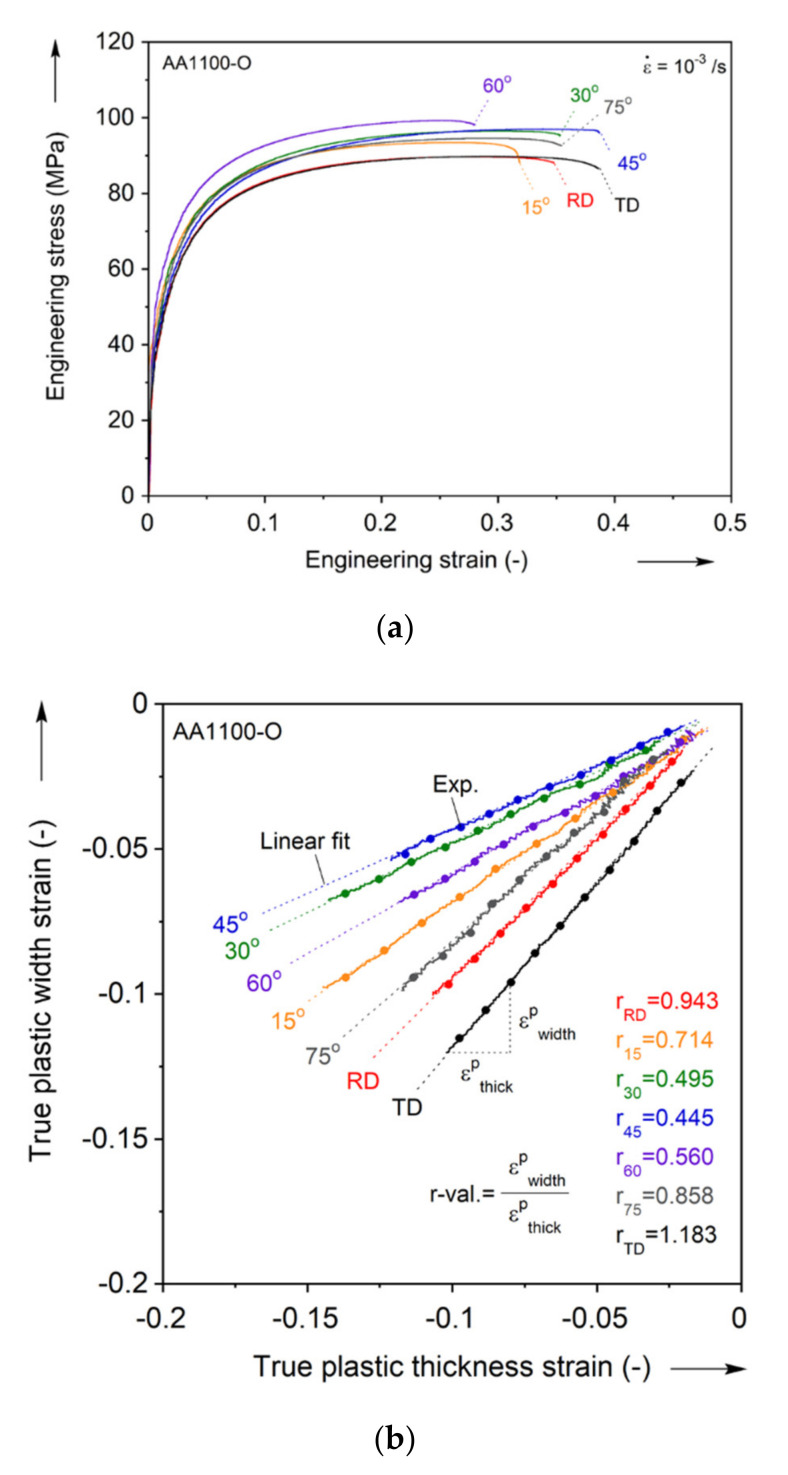
Experiment for uniaxial tension in every 15° from the RD: (**a**) engineering stress-strain curves and (**b**) linear fitting for r-values determination.

The engineering stress-strain curves from uniaxial tension in every 15° are shown in Figure 3a. The curves reveal that some amount of plastic anisotropy is present in this cold-rolled sheet, even after annealing. The corresponding plastic strain ratio in the width and thickness directions, so called the r-value, equivalently Lankford coefficient, i.e., $r=d\varepsilon_{w}^{p}/d\varepsilon_{t}^{p}$, is shown in Figure 3b. The r-values are calculated in an average sense during uniform plastic deformation: The two plastic strains in the width and thickness directions are almost linearly correlated. Thus, a slope of the linear fit for each direction is reported as the r-value.

Uniaxial tension in the RD is used as the reference hardening curve. Then, the normalized flow stresses with respect to the reference one, i.e., $\sigma /\sigma_{RD}$, are calculated at the plastic work of $W^{p}$ = 10 MJ/m^3^ (equivalent to ${\bar{\varepsilon }}^{p}$ = 0.13) based on the work equivalence, i.e., $dW^{p}=\bar{\sigma }\cdot d{\bar{\varepsilon }}^{p}=\sigma_{ij}\cdot d\varepsilon_{ij}^{p}$ [10]. The value of 10 MJ/m^3^ is chosen so that these normalized values become saturated with respect to further increase of the plastic work (see Appendix A). It should be noted that the normalized values of this material are found to vary at small values of the plastic work range (up to $W^{p}$= 3 MJ/m^3^, equivalent to ${\bar{\varepsilon }}^{p}$ = 0.05), while they saturate at larger values. The value of 10 MJ/m^3^ is well-within the saturated regime. Similar findings are reported in the authors’ previous work on AA6013 [65].

**Figure 4:**
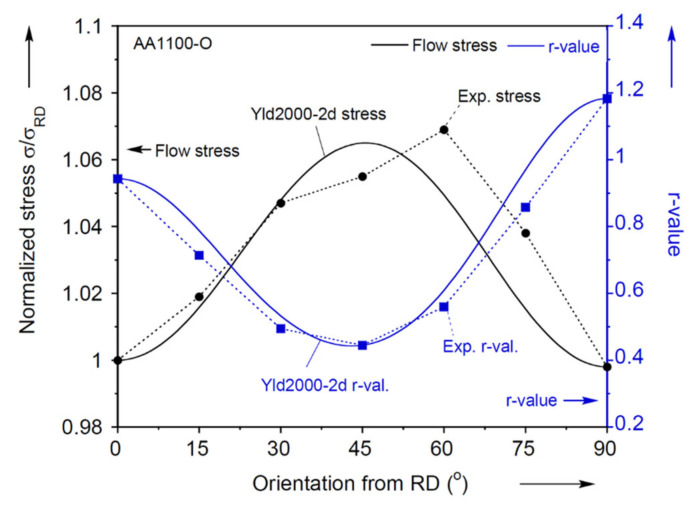
Anisotropy characterization in uniaxial tension in every 15° from the RD for flow stress and r-value. Prediction of Yld2000-2d is added to compare with the experiment.

The results for the plastic anisotropy characterization for the flow, yield and ultimate stresses and the r-values in the uniaxial tension experiments are summarized in Table 1 and Figure 4. This figure shows that the flow stress varies by about 7% between the different orientations. The r-value reveals clear anisotropy compared to the flow stress: the lowest is 0.445 at 45°, which is comparatively low (instead of 1 for isotropic plasticity), and implies a weak resistance to thinning in the thickness direction, resulting in lower formability.

#### 2.2.2. Plane-Strain Tension

**Figure 5:**
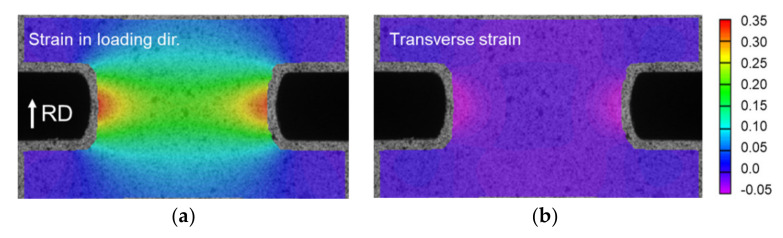
Strain fields measured during plane-strain tension. Strain in (**a**) loading and (**b**) transverse directions, showing zero strain conditions at central part of the test-section.

**Figure 6:**
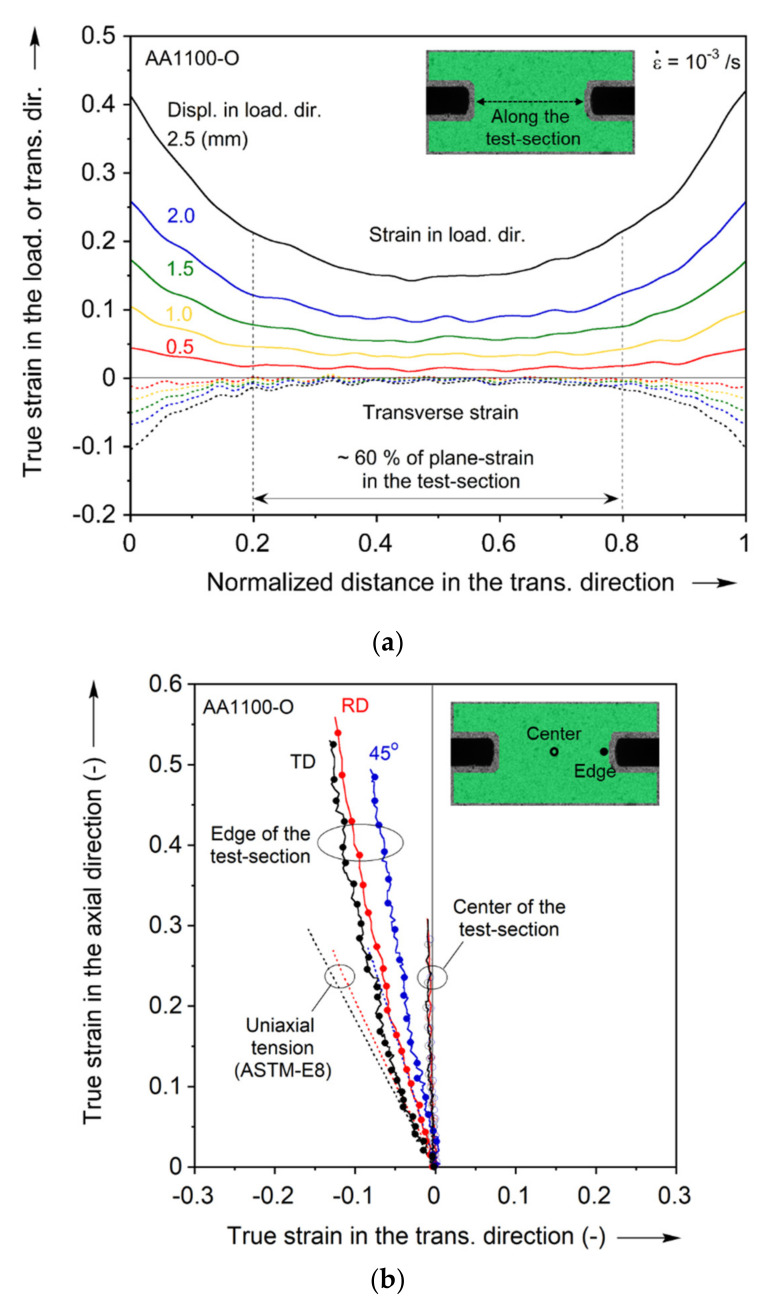
Experiment for plane-strain tension: (**a**) Variation of the loading and transverse direction strains along the test-section, and (**b**) strain paths probed at local material points near the edge and the center of the test-section.

The plane-strain tension experiments are performed in three orientations to the RD (see Figure 2a). These experiments provide three additional data points for calibrating the yield function, as described in the next section. Due to the specimen design, the wide shoulders remain elastic during loading and hence their lateral contraction is very limited. This imposes the desired constraint to the test-section, and hence the plane-strain deformation. The use of DIC revealed that indeed the transverse strain is very limited, as shown in the strain fields of Figure 5 and the plots of Figure 6. Figure 6a shows that the plane-strain deformation occurs throughout the deformation, and in the central 60% of the test-section. The strain paths shown in Figure 6b corroborate this performance of the specimen, for all orientations tested. Comparing the strain paths near the edges to the uniaxial tension ones reveals that they are quite close to each other, with differences recorded towards the end of the test due to lack of DIC correlation at the edge of the test-section.

Since the strain field is not uniform, the stress field will have a similar behavior. Hence, obtaining the stress as simply “force/area” only provides an average stress, which is not work-conjugate to the strains at the center of the test-section, as extracted from the DIC. The authors have proposed and applied on a large variety of materials a stress correction technique that is based on FE analysis [64,66,67,68]. Here, a FE model of 1/8 of the plane-strain tension specimen is created in Abaqus/Standard version 2018 (implicit solver), and meshed with quadratic, reduced integration elements (C3D20R). The stress field is compared with the average one, obtained from the macroscopically applied force. This procedure yields an average correction factor of 0.91, which is then applied to the average stress obtained from the experimental force and initial cross-sectional area. It should be noted that the lateral stress cannot be determined in this way. Instead, for the calibration of the yield function only the stress in the loading direction and the zero condition for the lateral (plastic) strain is used. The lateral stress is then obtained as an outcome of the calibration (see Section 2.3.1 for more details).

#### 2.2.3. Disk Compression

**Figure 7:**
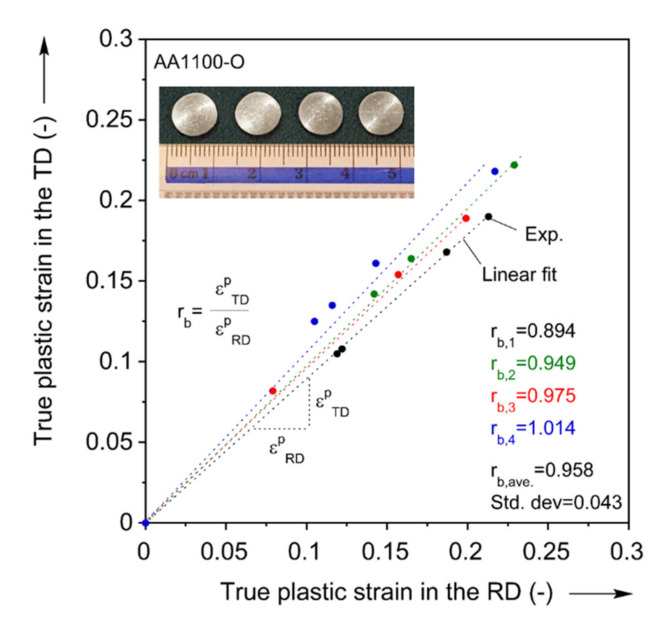
Experiment for disk compression and determination of $r_{b}$ value.

As the last plasticity characterization test, the plastic strain ratio of the RD and TD at the equibiaxial stress state, termed $r_{b}$, i.e., $r_{b}=\varepsilon_{TD}^{p}/\varepsilon_{RD}^{p}$, is determined using the disk compression experiment [27,69]. A well-lubricated, coin-shaped specimen of 8 mm diameter is compressed in the thickness direction using an Instron 1350 servo-hydraulic testing machine (Norwood, MA, USA) [66,67,70,71]. The experiment is interrupted at several levels of compressive thickness strain and in each step, the lengths along the RD and TD are measured manually using a micrometer. Then, the strain ratio at equibiaxial stress $r_{b}$ is determined by a slope of linear fit of the experimental data points as seen in Figure 7. The experiment is performed four times, and an average is taken for the yield function parameter calibration: the value is $r_{b}$ = 0.958 with standard deviation of 0.043. This deviation is slightly higher (but still acceptable) than the authors’other works, e.g., [65,71,72], possibly due to experimental errors caused by the thinner and softer material condition. This value is included in Table 1.

#### 2.2.4. Strain-Rate Sensitivity

**Figure 8:**
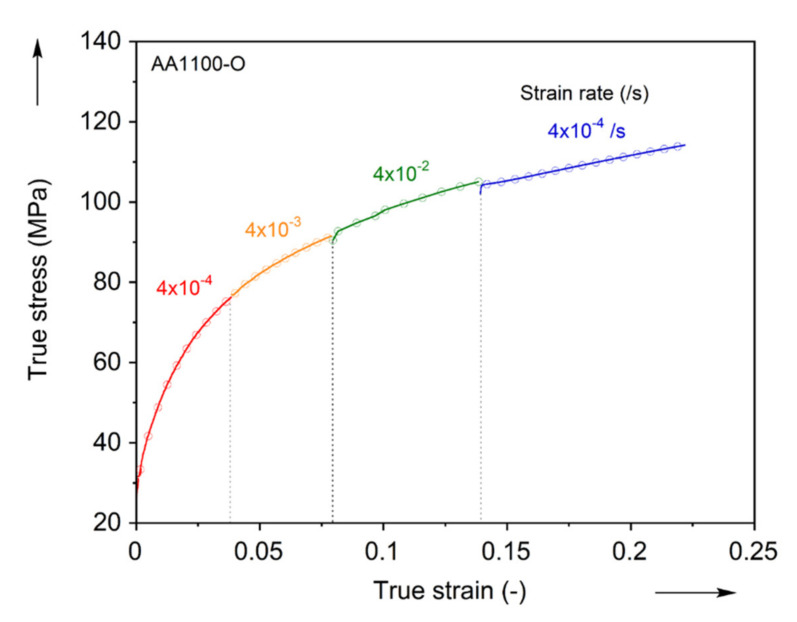
Jump test to measure the strain-rate dependence of AA1100-O.

The strain-rate sensitivity of the AA1100-O material is captured by the strain-rate-jump test [73,74,75,76]. During a uniaxial tension experiment, the crosshead velocity is suddenly changed, so that the strain-rate jumps first from 4 × 10^−4^/s to 10 times higher, then another 10 times higher, and then it drops back to 4 × 10^−4^/s (see Figure 8). This experiment is performed thrice. Assuming the strain-rate dependence of the material could be described with a power law, i.e., $\sigma =C{\dot{\varepsilon }}^{m}$, the average exponent m is determined (e.g., [73,75]) from the three jumps as between 0.001 and 0.008. This establishes the fact that the AA1100-O material can be considered as rate-insensitive, at least in the strain-rate range considered in this work. This finding is in concert with the literature [77], as well as earlier work of the authors in different aluminum alloys, e.g., [71,76].

### 2.3. Material Modeling for Plasticity

Based on the experiments in the previous section, the parameters of the material models are calibrated: plastic flow is assumed to be governed by a rate-independent, associated flow-rule; the plastic anisotropy is captured by a non-quadratic anisotropic yield function, i.e., Yld2000-2d [27]; and the stress-strain curve is extrapolated by a saturated-type isotropic hardening law, i.e., Voce.

#### 2.3.1. Anisotropic Yield Function

The anisotropic, non-quadratic yield function for plane-stress suggested by Barlat et al. [27], i.e., Yld2000-2d, is based on the isotropic, non-quadratic Hershey-Hosford yield function [78,79], which can be written as follows:

(1) $${\left|s_{1}-s_{2}\right|}^{n}\;+\;{\left|2s_{1}+s_{2}\right|}^{n}\;+\;{\left|s_{1}+2s_{2}\right|}^{n}=2\sigma_{o}^{n}$$

where the principal deviatoric stresses are $s_{1}$, $s_{2}$, and $s_{3}$ and the equivalent stress is $\sigma_{o}$. It is reformulated by tensors $X^{\prime }$ and $X^{\prime \prime }$ as:

(2) $${\left|X_{1}^{\prime }X_{2}^{\prime }\right|}^{n}\;+\;{\left|2X_{1}^{\prime \prime }+X_{2}^{\prime \prime }\right|}^{n}\;+\;{\left|X_{1}^{\prime \prime }+2X_{2}^{\prime \prime }\right|}^{n}=2\sigma_{o}^{n}$$

which are linearly transformed by $C^{\prime }$, $C^{\prime \prime }$, T, $L^{\prime }$ and $L^{\prime \prime }$ operators as

(3) $$X^{\prime }=C^{\prime }s=C^{\prime }T\sigma =L^{\prime }\sigma \;\mathrm{and}\;X^{\prime \prime }=C^{\prime \prime }s=C^{\prime \prime }T\sigma =L^{\prime \prime }\sigma$$

where $C^{\prime }$ and $C^{\prime \prime }$ are transformation tensors for deviatoric stress s to $X^{\prime }$ and $X^{\prime \prime }$, respectively, and $L^{\prime }$ and $L^{\prime \prime }$ for Cauchy stress σ. The matrices $L^{\prime }$ and $L^{\prime \prime }$ are composed of anisotropic material parameters $\alpha_{i,\;i=1\sim 8}$ as follows:

(4) $$\left[\begin{array}{c} L_{11}^{\prime }\\ L_{12}^{\prime }\\ \begin{array}{c} L_{21}^{\prime }\\ L_{22}^{\prime }\\ L_{66}^{\prime } \end{array} \end{array}\right]=\;\left[\begin{array}{ccc} 2/3 & 0 & 0\\ -1/3 & 0 & 0\\ 0 & -1/3 & 0\\ 0 & 2/3 & 0\\ 0 & 0 & 1 \end{array}\right]\;\left[\begin{array}{c} \alpha_{1}\\ \alpha_{2}\\ \alpha_{7} \end{array}\right]\;\left[\begin{array}{c} L_{11}^{\prime \prime }\\ L_{12}^{\prime \prime }\\ \begin{array}{c} L_{21}^{\prime \prime }\\ L_{22}^{\prime \prime }\\ L_{66}^{\prime \prime } \end{array} \end{array}\right]=\frac{1}{9}\;\left[\begin{array}{ccccc} -2 & 2 & \;8 & -2 & \;0\\ \;1 & -4 & -4 & \;4 & \;0\\ \;4 & -4 & -4 & \;1 & \;0\\ -2 & \;3 & \;2 & -2 & \;0\\ \;0 & \;0 & \;1 & \;0 & \;9 \end{array}\right]\;\left[\begin{array}{c} \alpha_{3}\\ \alpha_{4}\\ \alpha_{5}\\ \alpha_{6}\\ \alpha_{8} \end{array}\right]$$

**Figure 9:**
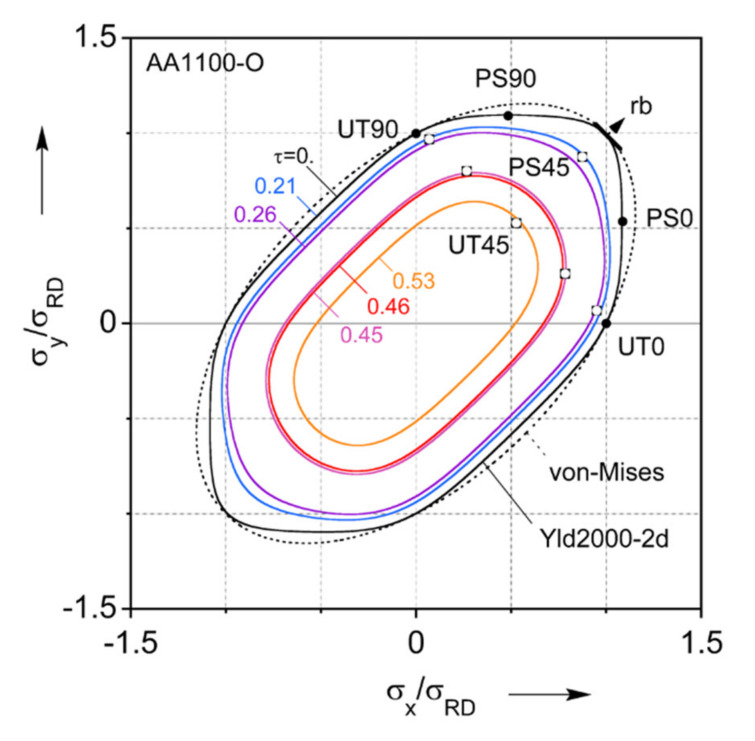
Yield loci predicted by Yld2000-2d (solid lines) and von-Mises (dashed line) with experiments (symbols) used for the parameter calibration.

The non-quadratic exponent n is a material parameter associated with the crystal structure [80], and n = 8 is set for this material as appropriate for face-centered cubic (FCC) crystals. For the parameter calibration of Yld2000-2d, the experiments described previously and summarized in Table 1 are used to calibrate 8 parameters,$\;\alpha_{i,\;\;i=1\sim 8}$, by using a nonlinear least-square method. The minor stress component of the plane-strain tension experiments is found by enforcing the corresponding plastic strain to be equal to 0 and using the associated flow-rule. The identified parameters are summarized in Table 2. Yield loci with iso-shear stresses are projected to the plane of τ = 0 MPa of Yld2000-2d and von Mises. Both of these criteria are plotted in Figure 9 along with the experiments used for the calibration. It should be noted that the empty symbols of the experiments indicate that they are not located on the projected plane. Their corresponding shear stresses are also mentioned in Figure 9. Furthermore, a comparison of the Yld2000-2d predictions of normalized flow stress and r-value in every 15° to the uniaxial tension experiments is included in Figure 4. Both plots clearly show that the Yld2000-2d captures the plastic anisotropy of this material much better than the von Mises isotropic yield function. See Appendix B for further comparisons between the two yield criteria considered. Material parameters for the Extra Deep Draw Quality (EDDQ) carrier blank are also included in Table 2.

#### 2.3.2. Strain Hardening Law

**Figure 10:**
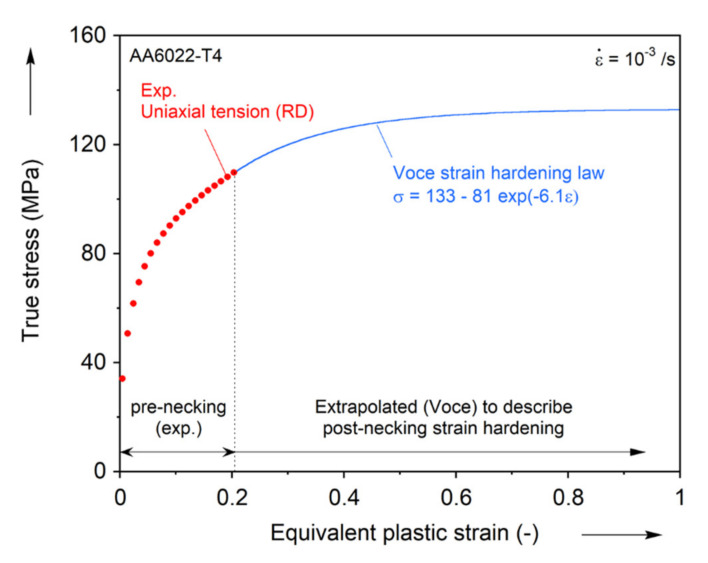
Extrapolation of hardening curve using the Voce hardening law.

As discussed earlier, the rate-dependence of AA1100-O is negligible in the range of strain-rates considered in this work. Furthermore, due to its high thermal conductivity and low amount of plastic work expended for deformation, the deformation-induced heating can also be neglected [81,82]. Hence, elaborate identification schemes of the hardening curve at large strains (e.g., [83]) can be omitted in this work. Instead, a simple extrapolation of a hardening curve commonly used for aluminum alloys will suffice. In this spirit, the flow stress-strain curve of Figure 10 is extrapolated using Voce model, i.e., $\bar{\sigma }=K-p\cdot \exp\left(-q\cdot \bar{\varepsilon }\right)$ [84]. The true stress-strain curve of the uniaxial tension in the RD is fitted up to the strain level $\bar{\varepsilon }$ = 0.2, which is the maximum uniform elongation, and the post-necking behavior is predicted by the calibrated parameters listed in Table 3.

## 3. Formability

### 3.1. Experimental Set-Up

The FLC of the AA1100-O is constructed using the Marciniak test [11,74,85], i.e., a flat-headed punch, a carrier sheet, and specimens with test-sections of different widths.

#### 3.1.1. Specimens

**Figure 11:**
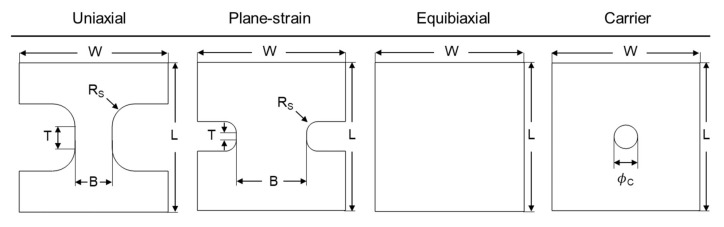
Geometries of the Marciniak specimens for all three strain paths and the carrier blank.

Three different specimen geometries are used, to probe uniaxial, plane-strain, and equibiaxial tension, respectively. Figure 11 shows the geometries, and Table 4 provides the detailed dimensions. The base length (L) and width (W) of all specimens are 200 × 200 mm^2^. These geometries are based on the ISO 12004-2 standard [44], with slight modifications to the fillet radius (R_S_) and length of test-section (termed “shaft length” in the ISO standard) (T) on the plane-strain specimen to ensure that fracture (rupture) occurs in the center of the specimen and not in the fillet radius. To assist with the strain localization in the center of the specimen, a carrier blank with a 32 mm hole diameter, made of EDDQ steel sheet of 1 mm thickness is placed between the specimen and the punch. The geometry of the carrier blank is included in Figure 11. All specimens are extracted along the RD. They are produced by waterjet machining, and the sides of the test-section are further manually polished with sandpaper.

#### 3.1.2. Equipment and Testing Procedure

**Figure 12:**
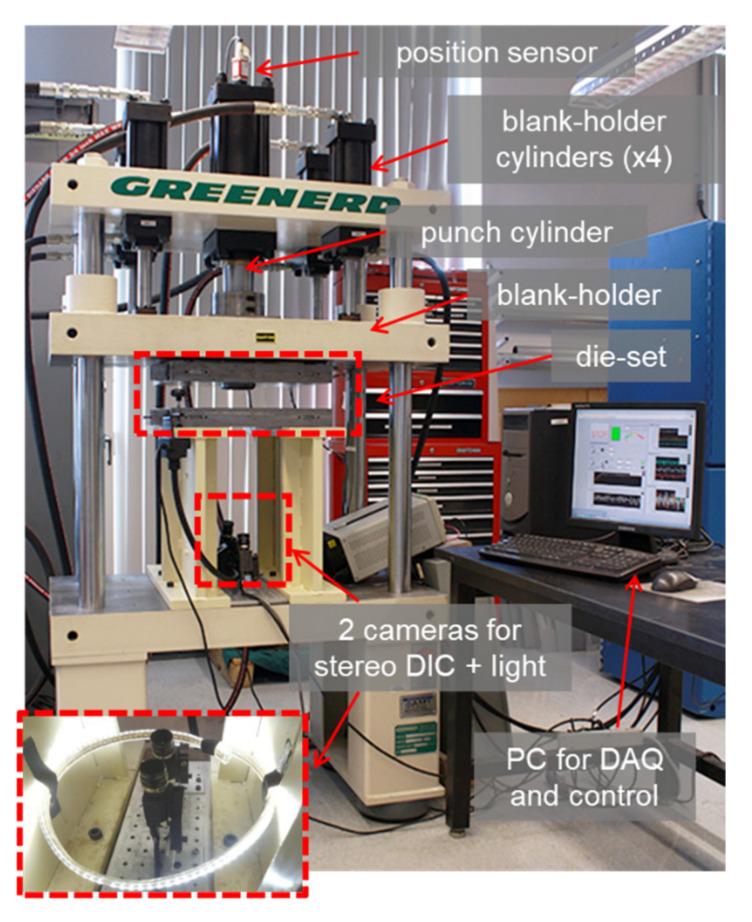
Instrumented hydraulic press used for Marciniak FLC experiments with its major components identified.

The FLC experiments are performed on a fully-instrumented Greenerd 260 kN hydraulic press with five hydraulic cylinders (the main one actuating the punch and remaining four the blank holder), see Figure 12. The press is equipped with load-cells to measure the punch and blank holding forces, as well as with a Linear Resistive Transducer (LRT) to provide the punch displacement. In earlier studies, it was shown that even though the punch is controlled with an open-loop system, for the forces and velocities considered in this work, the velocity is constant [64].

**Figure 13:**
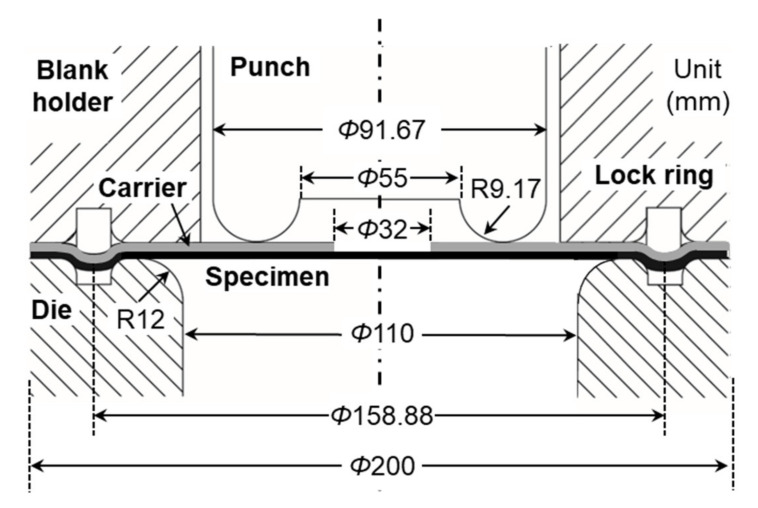
Cross-sectional view of the tooling used for Marciniak FLC experiments, showing the flat punch, die, blank holder, lock ring, and the specimen and carrier blanks.

The press tooling is designed based on the ISO 12004-2 standard [44] and shown in Figure 13. In order to reduce friction between the specimen and the tooling, Drawsol WM 4740 lubricant diluted with water in 3:1 proportions (water:oil) is applied generously to all interfaces, except between the specimen and carrier blank, where higher friction is desired. To measure strain in real-time during the experiment, a stereo-type Digital Image Correlation, i.e., 3D-DIC, system is used with two Grasshopper 2.0 MP FireWire (GRAS-20S4M-C, FLIR, Wilsonville, OR, USA) digital cameras with 1624 × 1224 pixel resolution and a rope LED light illuminating the specimen surface evenly. The entire setup of two cameras and the rope light under the press tooling is shown in a red box at the left bottom corner in Figure 12.

Both specimen and carrier blanks are centered in the tooling system and then they are tightly held by pressurizing the blank holder cylinders to 55 Bar (800 psi), which induces a blank-holding force of approximately 120 kN. In addition, a lock ring between the holder and the die is used to prevent the specimen from drawing into the die cavity (see Figure 13). Once the specimen is fully clamped in this way, the punch is lowered at a velocity of approximately 0.2 mm/s, while the DIC system acquires images every 0.5 s.

### 3.2. Experiments

**Figure 14:**
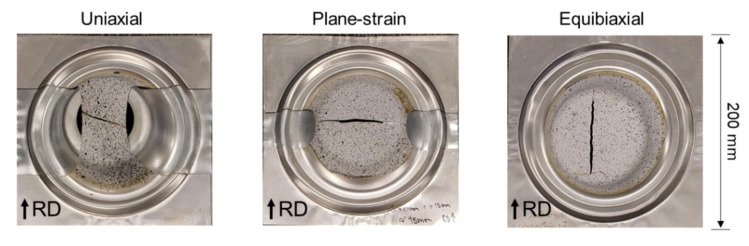
Photograph of the FLC specimens after fracture, for the three strain paths tested.

Marciniak-type FLC experiments are performed at least thrice with each specimen, so that the fracture initiates within the test-section, as seen in Figure 14. An iterative adjustment of the fillet radius (R_S_) and shaft length (T) (see Figure 11) was performed until that was accomplished, i.e., the fracture did not initiate from the fillet or the shoulder areas, for the plane-strain tension specimen.

**Figure 15:**
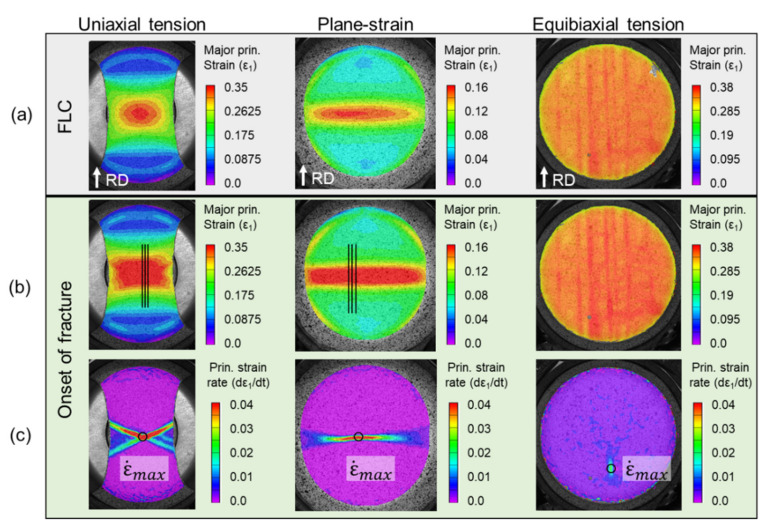
Contours of 1st principal strain during the FLC experiments (**a**) at FLC and (**b**) onset of fracture and (**c**) strain rate at onset of fracture. The lines and dots added to the images are explained in the text. Note that the scales are different, since each experiment deforms to different strain.

The DIC results allow the assessment of the full strain field throughout the deformation, as shown in Figure 15 for different instances during the tests (onset of necking, denoted as FLC, and onset of fracture). Both the uniaxial and plane-strain specimens show a neck forming perpendicular to the major loading direction. Upon closer inspection, the uniaxial specimen is beginning to form two angled bands of localized thinning, one of which will result in fracture (see Figure 14). It is interesting to note that the equibiaxial specimen forms a network of localization bands along the RD and TD. The bands along the RD are dominant, and indeed one of them will lead to fracture (as in Figure 14). Inspecting the corresponding strain-rate, i.e., the third row of snapshots in Figure 15, allows an easier observation of the localization process and physical location(s). This fact will be exploited for the detection of the limit (or necking) strains, as described later in this Section.

#### 3.2.1. Strain Paths

**Figure 16:**
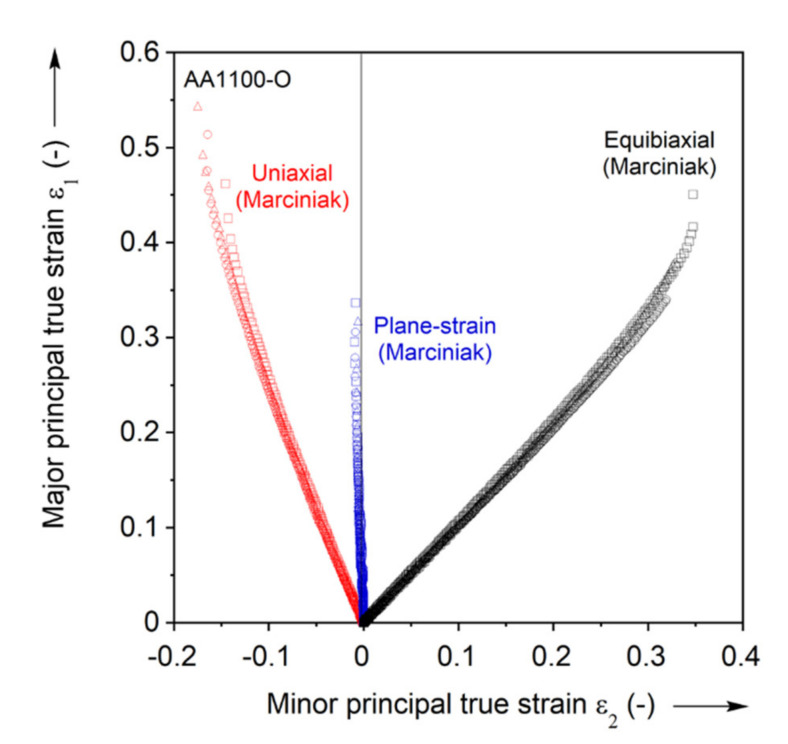
Strain paths recorded during the Marciniak FLC experiments. Three for each Marciniak experiment are included.

For each FLC experiment, the major and minor principal strains over the entire test-section of the specimen are calculated using the VIC-3D software with a subset of 17, step-size of 3, and filter of 15 pixels. These specific numbers are determined by parametric study with respect to the major principal true strain $\varepsilon_{1}$ (see Appendix C for details). The strain paths induced in each test-section, as well as the limit strains, i.e., the strains at the onset of necking, are determined as follows. The location where necking will initiate is first detected, from the strain field at the onset of fracture and the location that has the highest major principal strain-rate, see third row of Figure 15. Then, the major and minor principal strains are extracted from that location. Figure 16 shows the strain paths up to fracture of the Marciniak tests performed. The paths are highly linear until near the end of the test, as expected for the Marciniak procedure [74,85,86]. Towards the end of the test, the uniaxial and equibiaxial paths become more vertical, i.e., further increments in minor principal true strain tend to zero, indicating that the paths shift towards plane-strain tension. Repeatability is also very good, as the figure contains all experiments for the three paths probed. Furthermore, the paths line-up very well with the intended uniaxial, plane-strain and equibiaxial tension during the entire deformation up to necking.

#### 3.2.2. Detection of Necking

The major challenge in determining the limit strains using DIC is that the strain components continuously increase, without a clear sign of the onset of necking. Furthermore, more traditional approaches such as detecting a drop in the punch force-displacement curve are not sensitive enough to detect the onset of necking. Therefore, a variety of approaches have been proposed in the literature (some were reviewed in the Introduction). In this work, three methods are discussed: The first is the one described in the ISO 12004-2 standard [44]; the other two are based on the second time-derivative of the strain signals and the noise inherent in the strain signals.

##### ISO Method

**Figure 17:**
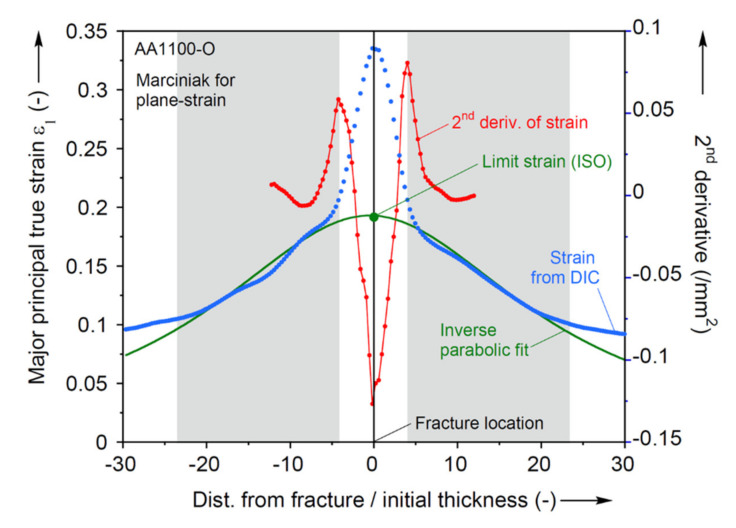
Determination of limit strain by ISO method.

In ISO 12004-2, it is suggested to use three 40 mm long lines, parallel to the specimen’s shaft and that intersect the fracture. These lines are visible in the second row of Figure 15. The major strain distribution along each of the intersection lines is read at the final image before fracture. An example of the maximum of three such readings is shown by the blue dots in Figure 17, which indicates that the maximum strain around the fracture is over three times higher than farther away from it. Furthermore, the strain gradient seems to extend laterally for about 10 times the initial sheet thickness, at the onset of fracture. Then, an inverse parabolic fit is created over two specific domains, or windows, on either side of the fracture (shown as grey boxes in Figure 17). To determine the size of these windows, the second spatial-derivative of the major true strain is plotted for 6 mm on either side of the highest strain recorded. The maximum points of the second derivative on each side of the fracture set the inner boundaries of the fitting windows. For the outer boundaries, the window width is calculated by the equation below [44]:

(5) $$w=10\cdot \left(1+{\bar{\varepsilon }}_{2}/{\bar{\varepsilon }}_{1}\right)$$

where w is the window width (in mm) and ${\bar{\varepsilon }}_{1}$ and ${\bar{\varepsilon }}_{2}$ are the averages of the major and minor strains at the two inner boundaries, respectively. With the inner and the outer boundaries known (and shown as the gray areas in Figure 17), the inverse parabolic curve fitted to the major strain data in the two fitting windows is:

(6) $$f\left(x\right)=1/\left(ax^{2}+bx+c\right)$$

where *x* is the length of the specimen in mm; and a, b, and c are the coefficients that are adjusted to achieve the best fit approximation. The point of fit that coincides with the location of fracture on the specimen is set as the major limit strain. To determine the second derivative of the major strain, the method proposed in the ISO 12004-2 was used at first; however, this was found to not always be reliable, making it hard to determine the inner boundaries. Instead, a central-difference method is used to calculate the second derivative in this work.

In the ISO 12004-2 method, the minor limit strain is calculated indirectly by using the thickness strain. The thickness strain is calculated from the major and minor strain values from the intersection lines. By using the same fit windows that are used to determine the major limit strain, a new inverse parabolic fit is created, and the thickness limit strain is found. Using the major and thickness limit strains, the minor limit strain is finally determined.

**Figure 18:**
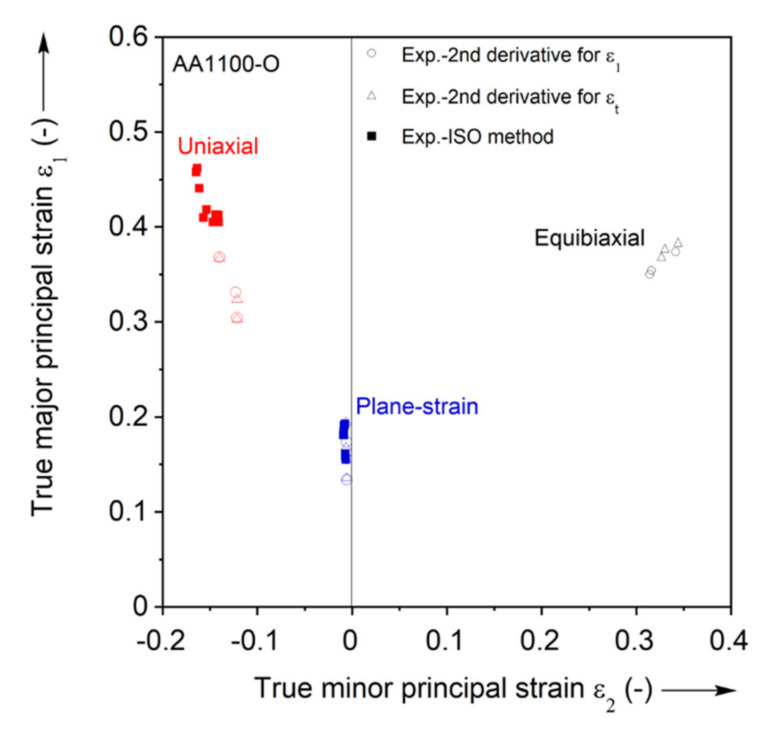
FLC points identified by second derivative and ISO methods and FEA.

The ISO12004-2 method is utilized for both the uniaxial and plane-strain case but not for the equibiaxial case. This is because multiple necks appear on these specimens, see Figure 15. The standard describes that it is not applicable in such cases [44]. The results of the ISO method for the uniaxial and plane-strain tension cases are shown in the FLC of Figure 18 as solid squares.

##### Second Derivative Methods

**Figure 19:**
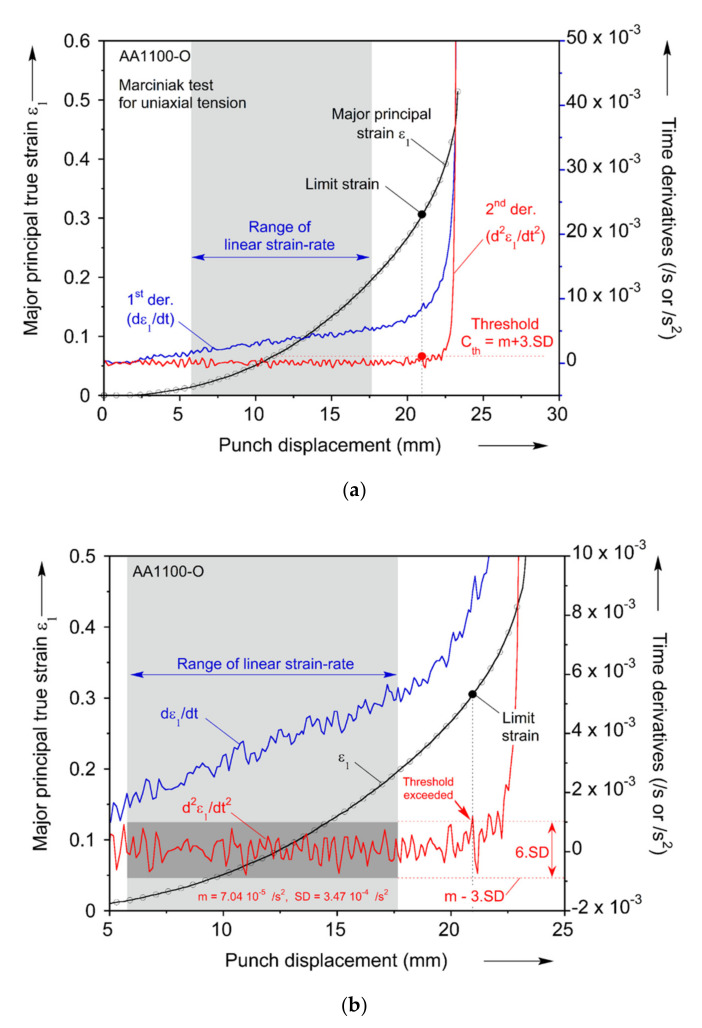
Determination of FLC points by (**a**) the second time-derivative method and (**b**) its close-up near the limit strain.

The other two methods used to determine the FLD in this work are based on taking time-derivatives of the strain. The first and second time-derivatives of the major principal strain at the point where it has the highest strain-rate right before fracture (see third row in Figure 15) are computed using a central-difference method. In these computations, the raw data extracted from DIC is used since it was found that smoothing the data caused the necking point to be undetectable. It is then assumed that necking initiates when the second derivative of the major principal strain starts to increase beyond a threshold value $C_{th}$. At that moment, it is observed that the first derivative also increases precipitously. To determine the threshold value $C_{th}$, the average ($\bar{m}$) and the standard deviation (SD) of the second derivative is calculated in the largest possible region where the strain-rate (i.e., the first derivative) has a constant slope. By summing the average and three times of the standard deviation, the threshold value $C_{th}$ is determined, i.e., $C_{th}=\bar{m}\;+\;3\cdot SD$. The moment at which the second derivative passes above the threshold is identified as the initiation of necking. Figure 19 illustrates the details of this procedure. In particular, Figure 19b highlights with a light grey box the region of linear strain-rate, and with a dark grey box the corresponding region of the second derivative, from where $\bar{m}$ and SD are computed. Beyond that, the second derivative remains within the threshold $C_{th}$ until a punch displacement of about 21 mm, where it first exceeds $C_{th}$. This is then recorded as the onset of necking and the corresponding limit strain.

**Figure 20:**
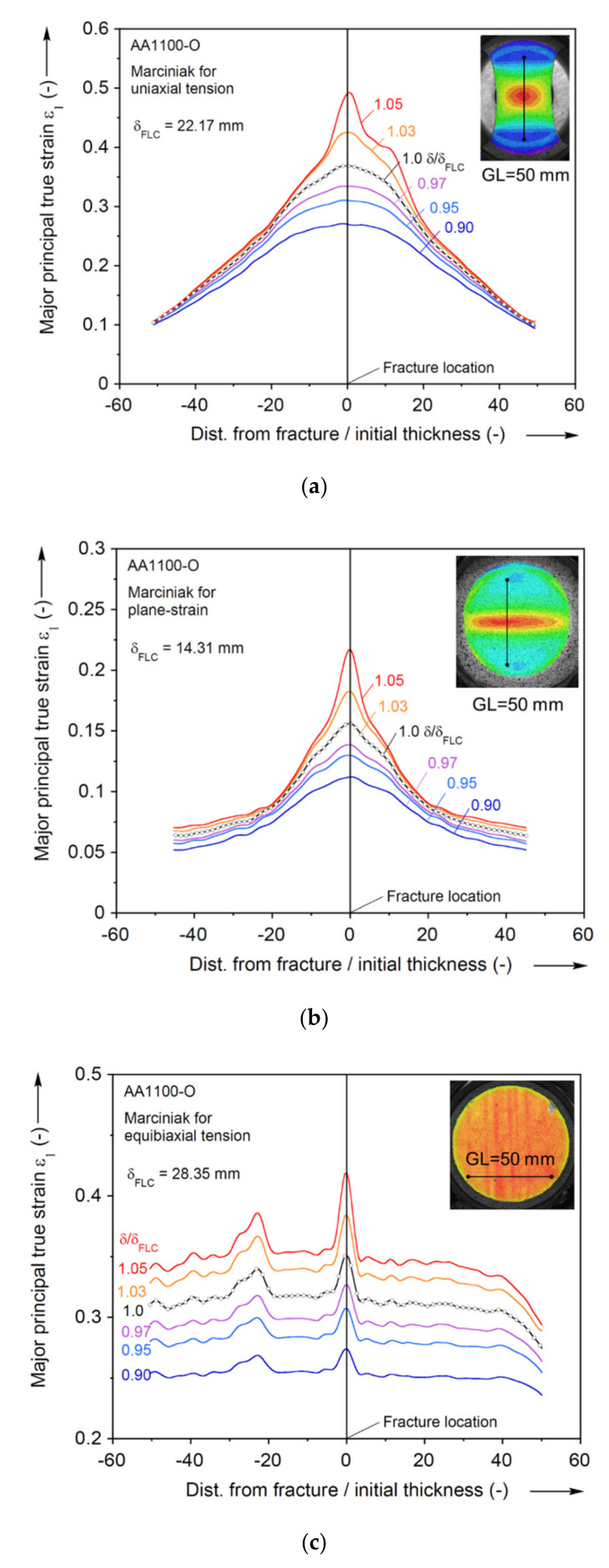
Strain profile near FLC limit identified by second derivative method of major principal strain for (**a**) uniaxial tension, (**b**) plane-strain tension, and (**c**) equibiaxial tension. The strain axes are in different scales, appropriate to each experiment.

While mathematically straightforward, it is interesting to explore whether this procedure has a physical meaning, i.e., whether it indeed identifies the onset of necking, and if so, how accurately. To that end, Figure 20 represents the evolution of a major principal strain for the three strain paths considered in this work. The strain profiles are retrieved along lines of 50 mm initial length (or 100 times the sheet thickness) and perpendicular to the fracture, i.e., ±25 mm from where the fracture initiated. These lines are included in the insets of Figure 20. The strain profiles shown are near the onset of necking, as identified by the second derivative of the major principal strain. In particular, the strain profiles are extracted at several punch displacements (δ) relative to FLC displacement ($\delta_{FLC}$), i.e., 0.9, 0.95, 0.97, 1.0 ($\delta_{FLC}$), 1.03, and 1.05 $\delta /\delta_{FLC}$. As Figure 20 indicates, strain clearly localizes in the center after $\delta_{FLC}$ in the uniaxial tension and plane-strain Marciniak experiments, while in the equibiaxial tension it appears that this happens about 2–3% of normalized punch displacement later. The localization spreads over a zone about 10 initial thicknesses wide, in which a strong strain gradient prevails. At any rate, it can be concluded that using the second derivative and threshold as described in this work identifies the onset of necking quite well, i.e., within a very few percent of the punch displacement that it occurs.

The second derivative method is applied for both major principal and thickness strains of the three Marciniak tests separately, and the FLC points obtained is included in Figure 18. Both show similar results, but the method for thickness strain presents slightly higher FLC points than that for major principal strain. The identified FLC values by the second derivative and ISO methods are comparable in plane-strain, while the ISO method is almost 30% higher than the second derivative in uniaxial tension.

## 4. Finite Element Validation of Material Model

To validate the material model calibrated in Section 2.3, FE models of the Marciniak FLC experiments are created in Abaqus/Standard ver. 2018 (implicit solver). The constitutive models described in Section 2.3 for the non-quadratic anisotropic yield function, i.e., Yld2000-2d, and isotropic strain hardening, i.e., Voce law, are implemented in a user-material subroutine (UMAT) using the parameters listed in Table 2.

**Figure 21:**
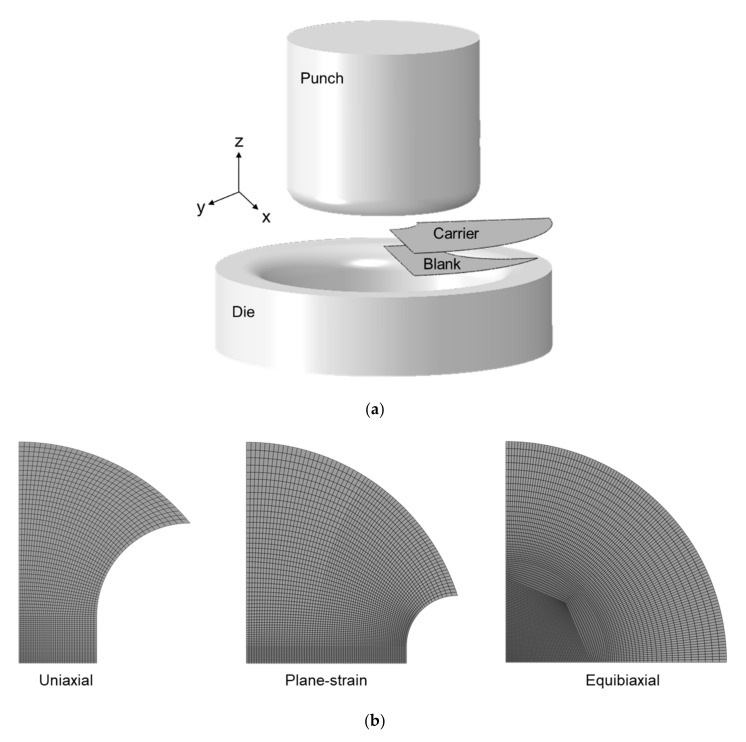
(**a**) FE model components for Marciniak simulation and (**b**) mesh design of blanks for uniaxial tension, plane-strain, and equibiaxial tension. Finer mesh is concentrated in the region where deformation is concentrated during deformation.

Taking advantage of the symmetries present, quarter models of the specimen and carrier blanks are constructed, using reduced-integration shell elements (S4R) of 0.5 × 0.5 mm^2^ and 2.0 × 2.0 mm^2^ size at the minimum, respectively. The die, holder and punch are modeled as analytical rigid bodies. To simulate the lock ring in the experimental setup, the elements at the edge of the specimen and carrier blanks are fully-constrained, so that no material flows through the lock ring as the specimen is deformed. Figure 21a shows the FE model used for the Marciniak simulation and Figure 21b for the mesh design of each Marciniak specimen types, i.e., uniaxial tension, plane-strain, and equibiaxial tension. The friction coefficient is assumed to be 0.47 [87] for the dry surface between the blank and carrier blank and 0.1 for lubricated surfaces.

**Figure 22:**
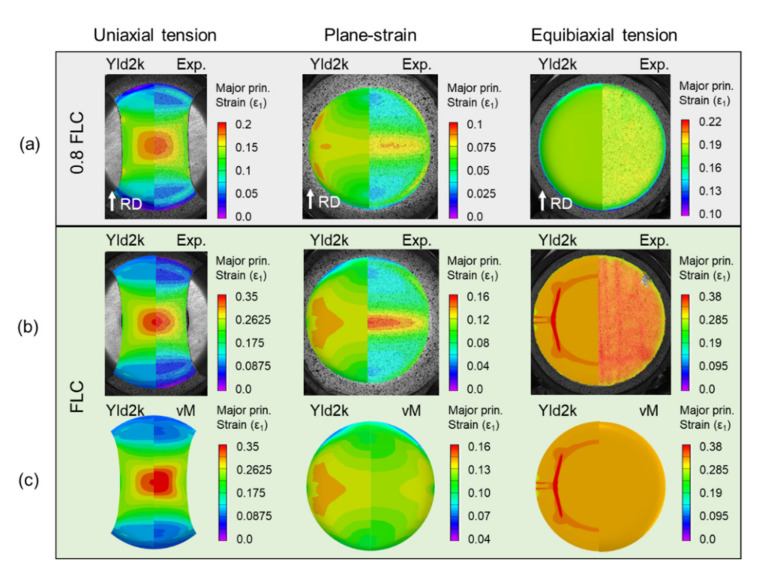
Contours of major principal strain for the FLC experiments and predictions (**a**) at 0.8 FLC and (**b**) FLC, and (**c**) comparison of Yld2000-2d and von Mises at FLC. The strain levels are different, as appropriate in each case.

Figure 22 shows comparisons of the experiment and simulations for major principal strain distribution at 0.8 of FLC (0.8 $\delta /\delta_{FLC}$) and FLC, i.e., onset of necking ($\delta_{FLC}$). For 0.8 FLC, all three predictions of Yld2000-2d show good agreement with the experiments, which means that the material models incorporated in this study capture the elasto-plastic behavior very well up to this range of plastic deformation. However, at the onset of necking, the predictions of both Yld2000-2d and von Mises are a bit erroneous in plane-strain because the strain is rather prone to be localized near the punch radius instead of the center. In contrast, for uniaxial tension, Yld2000-2d captures the experimental strain field well, while von Mises overpredicts the localized region. This indicates that neglecting the plastic anisotropy in the modeling can increase the prediction error. Equibiaxial tension is predicted reasonably well by both Yld2000-2d and von Mises in terms of average strain at the onset of necking although only Yld2000-2d illustrates localized strain as a vertical band, which is similarly observed in the experiment.

**Figure 23:**
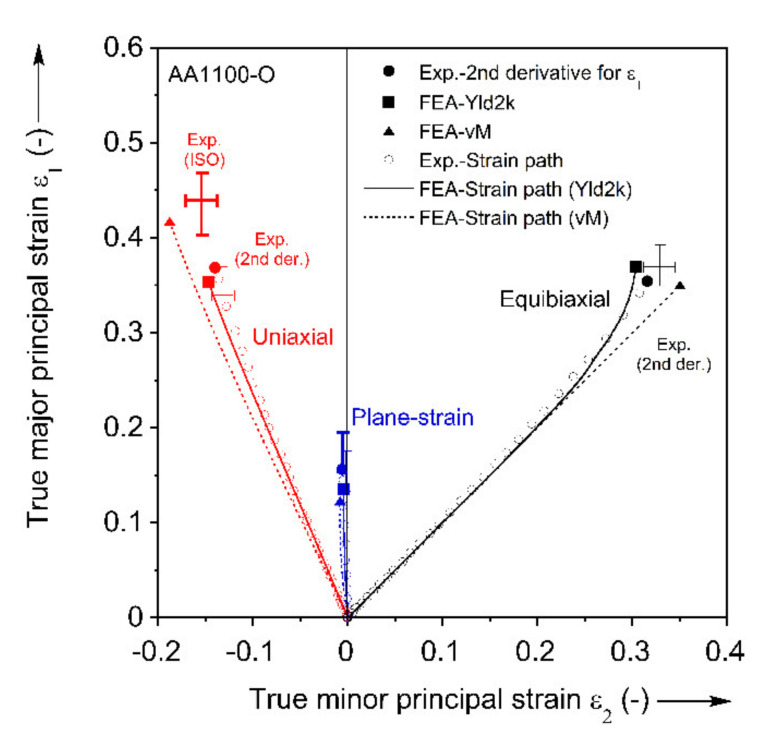
Comparison of experimental and predicted strain paths. Also shown are the experimental FLC points and associated error bars.

Figure 23 shows the experimental FLC points from the second derivative method and FE model data from the element of max. strain extracted up to this same punch displacement. Only the second derivative method is utilized to compare with the prediction because the ISO method is not applicable for the equibiaxial tension due to the fact that multiple necks appeared on the specimen surface (as mentioned earlier in Section 3.2.2. ISO method). The replicated experiments are presented by error bars both for ISO and the second derivative methods. For uniaxial tension, Yld2000-2d predicts the limit strain and the strain path reasonably well, and within the error bar of the second derivative method. In contrast, von Mises overpredicts the limit strain from the second derivative method, but is close to the strain determined by the ISO method. However, the strain path predicted by von Mises is clearly different from the experiment, which is expected because of the anisotropy of the material (see r-values in Table 1). Both models predict the strain state well for plane-strain, despite the contour plot concerns noted in Figure 22. Finally, for the equibiaxial case, both yield criteria predict similar strain paths during the initial deformation, but Yld2000-2d starts localizing and turning to a plane-strain path earlier than von Mises. As a result, the limit strain of Yld2000-2d is closer to the experiment, near the error bar of the second derivative method, compared to von Mises. It should be also noted that a reason for the similar strain path of Yld2000-2d and von Mises is driven by the in-plane anisotropy, $r_{b}\;$= 0.958 from disk compression in Table 1, which is close to the isotropic value of 1.

## 5. Conclusions

The plasticity and formability of an annealed, cold-rolled thin sheet of commercially-pure aluminum is assessed in this work using a combination of experiments and analysis. The work includes two parts, first the constitutive modeling and then the determination of the FLC points. In the first part of the work, the plastic anisotropy of AA1100-O is assessed by uniaxial and plane-strain tension, and disk compression experiments. These results are used to calibrate the Yld2000-2d anisotropic yield criterion and the Voce hardening curve. The FLD is determined by Marciniak-type experiments under uniaxial, plane-strain, and equibiaxial tension. The strain paths and limit strains are assessed through DIC. For the limit strains, the method proposed by ISO, as well as two methods based on the second derivative of strain are examined. Note that due to multiple necking locations, the ISO method is not used for the equibiaxial case. Finally, the FLC experiments are simulated in order to validate the plastic anisotropy and constitutive modeling.

The major observations and findings from this work are:

(1) The material is clearly plastically anisotropic: The flow curves in uniaxial tension vary by about 7% between different orientations, and the r-values range from 0.45 to 1.18.
(2) The strain-rate sensitivity of AA1100-O is negligible in the quasistatic rates examined here.
(3) The Yld2000-2d anisotropic yield criterion captures the material characterization experiments very well, and much better than von Mises with respect to the final yield loci. This provided a contrast in the modeling with and without the consideration of anisotropy.
(4) Fracture in the FLC experiments always occurs well inside the test-section. However, accomplishing this for the plane-strain specimen requires significant iterations on the specimen geometry.
(5) The strain paths recorded are linear, and the AA1100-O material is seen to possess good formability.
(6) The FE model with the Yld2000-2d anisotropic yield criterion captures the experimental strain contour plots well for the uniaxial and equibiaxial cases and much better than the von Mises. The experimental plane-strain case is not predicted well by the FE simulations using either yield criteria for the onset of necking. On the other hand, the agreement is good for the majority of pre-necking deformation.
(7) The final strain state values for both experiments and FE simulations at the same punch displacement, which is set by the experimental second derivative method, are reasonably accurate for FE models with both yield criteria, but the Yld2000-2d model is better.

This work validates a computationally-efficient constitutive model and its ability to predict plastic flow during forming. This model can then be confidently used for stamping process design, whether in a conventional or IIoT-enabled environment.

## Figures and Tables

**Table 1:** Summary of mechanical properties for elasticity and plasticity ($W^{p}$ = 10 MJ/m^3^).

Young’s Modulus		E = 70 GPa	Poisson’s Ratio			ν = 0.3		
		RD	15°	30°	45°	60°	75°	90°
UT	$\sigma_{y}$ [MPa] _0.2 % offset_	23	27	28	30	32	30	26
	$\sigma_{UTS}$ [MPa]	90	93	97	97	99	95	90
	$\sigma /\bar{\sigma }$ (Standard deviation)	1.000	1.019 (0.006)	1.047 (0.002)	1.055 (0.007)	1.069 (0.01)	1.038 (0.009)	0.998 (0.007)
	r-value	0.943	0.714	0.495	0.445	0.560	0.858	1.183
PST	$\sigma /\bar{\sigma }$	**RD**		**45°**		**90°**		
		1.087		1.089		1.091		
DC	r_b_	0.958						
Jump	m	0.003–0.008						

**Table 2:** Parameter calibration of non-quadratic anisotropic yield function (Yld2000-2d).

Mat.	n	$\alpha_{1}$	$\alpha_{2}$	$\alpha_{3}$	$\alpha_{4}$	$\alpha_{5}$	$\alpha_{6}$	$\alpha_{7}$	$\alpha_{8}$
AA1100-O	8	0.969	1.041	1.025	0.995	1.006	1.003	0.891	0.943
EDDQ	6	0.962	1.119	0.891	0.912	0.929	0.762	0.999	1.011

**Table 3:** Parameter calibration of isotropic strain hardening model (Voce: $\bar{\sigma }=K-p\cdot \exp\left(-q\cdot \bar{\varepsilon }\right)$

K	p	q
133 MPa	81 MPa	6.1

**Table 4:** Detailed dimensions for each FLC specimen in mm.

Specimen	W	L	B	T	Rs	*ϕ*_c_
Uniaxial	200	200	50	30	30	-
Plane-strain	200	200	95	10	15	-
Equibiaxial	200	200	-	-	-	-
Carrier	200	200	-	-	-	32

**Figure A1:**
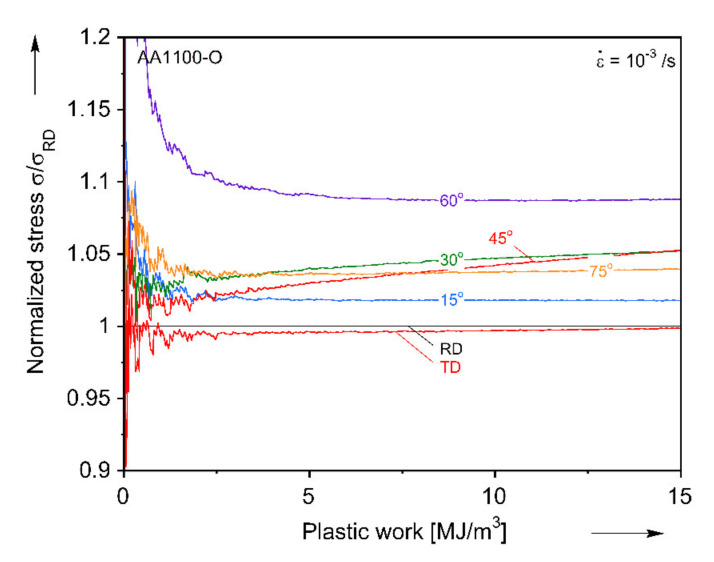
Evolution of normalized stress with plastic work.

**Figure A2:**
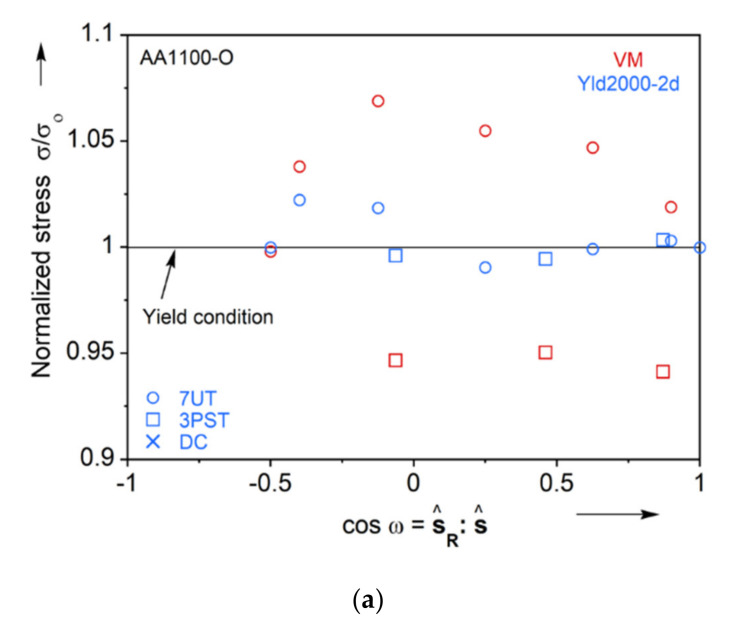

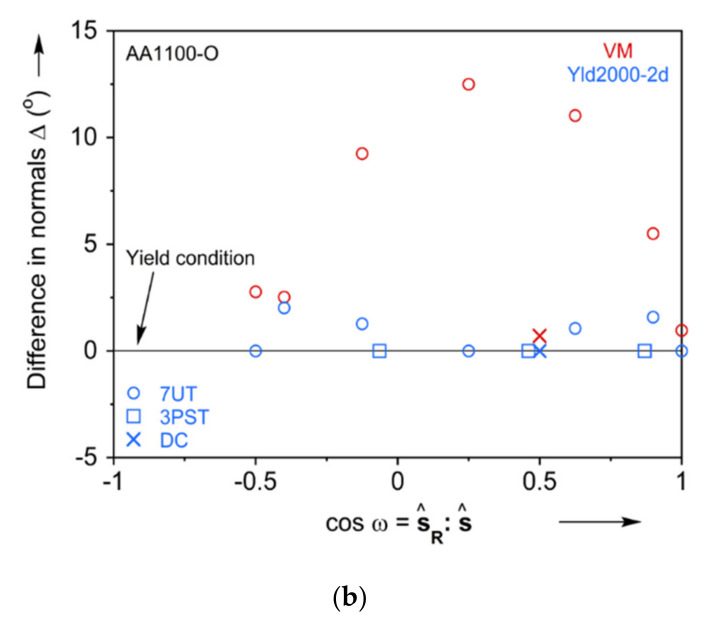
KBK anisotropy evaluation of (**a**) Yld2000-2d and (**b**) von Mises models.

**Figure A3:**
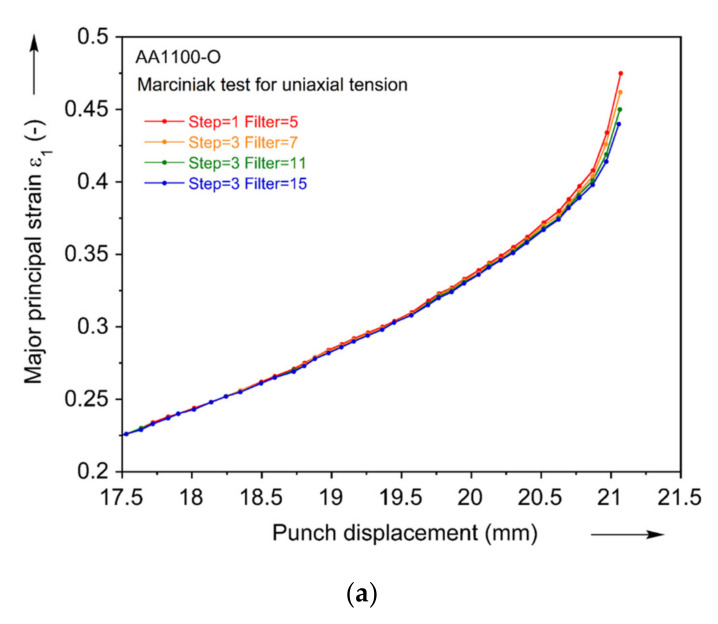

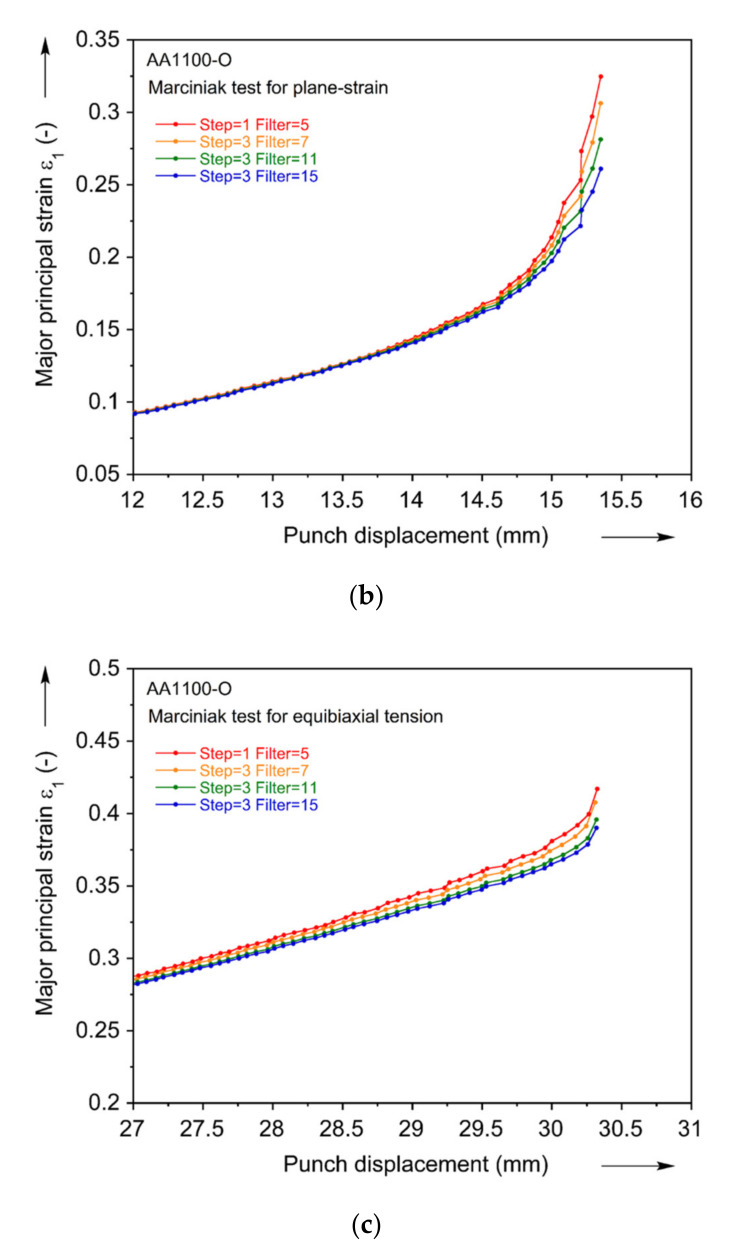
DIC parameter optimization for major principal strains for (**a**) uniaxial tension, (**b**) plane-strain, and (**c**) equibiaxial tension of Marciniak tests.

## Abbreviations

The abbreviations used in this work are tabulated below.

DC	Disk compression
DIC	Digital Image Correlation
GL	Gauge length
FCC	Face-centered cubic
FLC	Forming Limit Curve
FE	Finite elements
ND	Normal direction
PST	Plane-strain tension
RD	Rolling direction
TD	Transverse direction
UMAT	User-material (subroutine)
UT	Uniaxial tension

## Appendix A

The evolution of the ratio of the stress over the equivalent stress during plastic deformation is plotted in Figure A1. This establishes the fact that at a plastic work of $W^{p}$ = 10 MJ/m^3^ (equivalent to $\bar{\varepsilon }$ = 0.13) the evolution has reached saturation. This is the value used for calibrating the anisotropic yield function.

## Appendix B

The plastic anisotropy of AA1100-O is described in Section 2 and Figure 2, Figure 3, Figure 4, Figure 5, Figure 6, Figure 7, Figure 8 and Figure 9. An alternative way that provides similar information but in a more compact format is by representing them as proposed in [88]. The resulting pair of plots is given in Figure A2a,b. It can be seen that the Yld2000-2d captures the experimental stress values much better than the von Mises, both for the in-plane (i.e., zero shear) and out-of-plane (i.e., non-zero shear) states. Similarly, for the strains (or normal to the yield surface, i.e., Δ, in Figure A2), while von Mises is sometimes over 10° away from the experiment, the Yld2000-2d predictions are in every case within 2.5° of the experiments. These observations justify the additional investment in calibrating and implementing more advanced yield criterion, instead of resorting to classical J_2_ plasticity. Similar examples for other materials can be found in [65,66,67,71,72].

## Appendix C

When the spatial deformation is not uniform, the strain field can be sensitive to the DIC parameters used for the post-processing of images. In this regard, step and filter sizes can be the most interesting parameters. Reducing these values generally increases the spatial resolution, thus more accurately capturing a gradient. But on the other hand, it also increases the noise level. In order to identify the correct FLC value, the optimal parameters must be determined. Three parameter sets are compared with the base case (i.e., step of 1 and filter of 5 pixels), which is the condition with the highest spatial resolution. This is done for Marciniak tests in uniaxial, plane-strain, and equibiaxial tension. The evolution of the major principal strain with the punch displacement is plotted up to the onset of fracture for all three cases as seen in Figure A3a–c, for the four sets or DIC parameters. The uniaxial tension seems to be the least sensitive to the DIC parameters, and the equibiaxial one the most. Since the objective of this work is the detection of the limit strains (i.e., necking) and not the fracture ones, it can be concluded that the sensitivity is quite limited. For consistency, in this work the step of 3 and filter of 7 pixels are selected and used for the DIC post-processing of the all Marciniak experiments. This ensures that the spatial resolution of the strain gradients, as well as the level of noise is as comparable as possible among the different experiments that populate the FLD of Figure 18.
